# Understanding barriers and facilitators to clinic attendance and medication adherence among adults with hypertensive urgency in Tanzania

**DOI:** 10.1371/journal.pgph.0000919

**Published:** 2022-08-23

**Authors:** Godfrey A. Kisigo, Onike C. Mcharo, John L. Robert, Robert N. Peck, Radhika Sundararajan, Elialilia S. Okello

**Affiliations:** 1 Mwanza Intervention Trials Unit, National Institute for Medical Research, Mwanza, Tanzania; 2 Center for Global Health, Weill Cornell Medicine, New York, New York, United States of America; 3 Department of Emergency Medicine, Weill Cornell Medicine, New York, New York, United States of America; University of Global Health Equity, RWANDA

## Abstract

Hypertensive urgency is a major risk factor for cardiovascular events and premature deaths. Lack of medication adherence is associated with poor health outcomes among patients with hypertensive urgency in resource-limited settings. To inform the development of tailored interventions to improve health outcomes in this population, this study aimed at understanding facilitators and barriers to clinic attendance and medication adherence among Tanzanian adults with hypertensive urgency. We conducted in-depth interviews with 38 purposively selected participants from three groups: 1) patients with hypertension attending hypertension clinic, 2) patients with hypertension not attending hypertension clinic, and 3) clinic health workers. Interviews were conducted using a semi-structured guide which included open-ended questions with prompts to encourage detailed responses. In their narrative, patients and healthcare workers discussed 21 types of barriers/facilitators to clinic attendance and medication adherence: 12 common to both behaviors (traditional medicine, knowledge and awareness, stigma, social support, insurance, reminder cues, symptoms, self-efficacy, peer support, specialized care, social services, religious beliefs); 6 distinct to clinic attendance (transport, clinic location, appointment, patient-provider interaction, service fragmentation, quality of care); and 3 distinct to medication adherence (drug stock, side effects, medicine beliefs). The majority of identified barriers/facilitators overlap between clinic attendance and medication adherence. The identified barriers may be surmountable using tailored supportive intervention approaches, such as peer counselors, to help patients overcome social challenges of clinic attendance and medication adherence.

## Introduction

Hypertension affects nearly 1 billion adults worldwide and is the single most significant risk factor for premature mortality globally [1, 2]. In Tanzania, the prevalence of hypertension is high in both urban and rural areas [3], but only 20% of Tanzanian adults with hypertension are aware of their diagnosis, and among these, less than 1% are controlled [4]. As a result, adults with untreated or uncontrolled hypertension develop complications, including hypertensive urgency [5]—defined as a blood pressure >180/120 mmHg without new or progressive target organ damage. This complication is a major risk factor for cardiovascular events and mortality [6, 7].

In Tanzania, we have documented that 20% of patients with hypertensive urgency were hospitalized and 26% died within 6 months of being seen in the outpatient clinic [8]. Participants at the highest risk of poor outcomes were those who self-reported non-adherence to antihypertensive medications at the time of enrollment. These findings highlighted the urgent need for tailored intervention to improve clinic attendance and medication adherence so as to improve long-term outcomes in patients with hypertensive urgency [9].

However, evidence to inform interventions to improve hypertension treatments among adults presenting with hypertensive urgency is lacking. Several studies have elicited barriers and enablers to hypertension care in Sub-Saharan Africa (SSA), but none focused on patients with hypertensive urgency [10–14]. We need to better understand the perspective of patients with hypertensive urgency in Africa and their healthcare providers in order to design targeted interventions to help patients with hypertensive urgency. Therefore, this study aimed at understanding factors that drive and inhibit clinic attendance and medication adherence among Tanzanian adults with hypertensive urgency as reported by patients and healthcare providers.

## Methods

### Overview

This study was part of formative research to inform intervention design for adult patients with hypertensive urgency attending outpatient clinic in Mwanza, Tanzania. The study purposively selected 38 individuals relevant to outpatient care of hypertensive urgency to complete in-depth interviews. The development of semi-structured interview guides and qualitative data analysis were guided by the Andersen’s Behavioral Model of Health Services Use (ABMHSU) [15], which is introduced succinctly below.

### Theoretical framework

**Fig 1:**
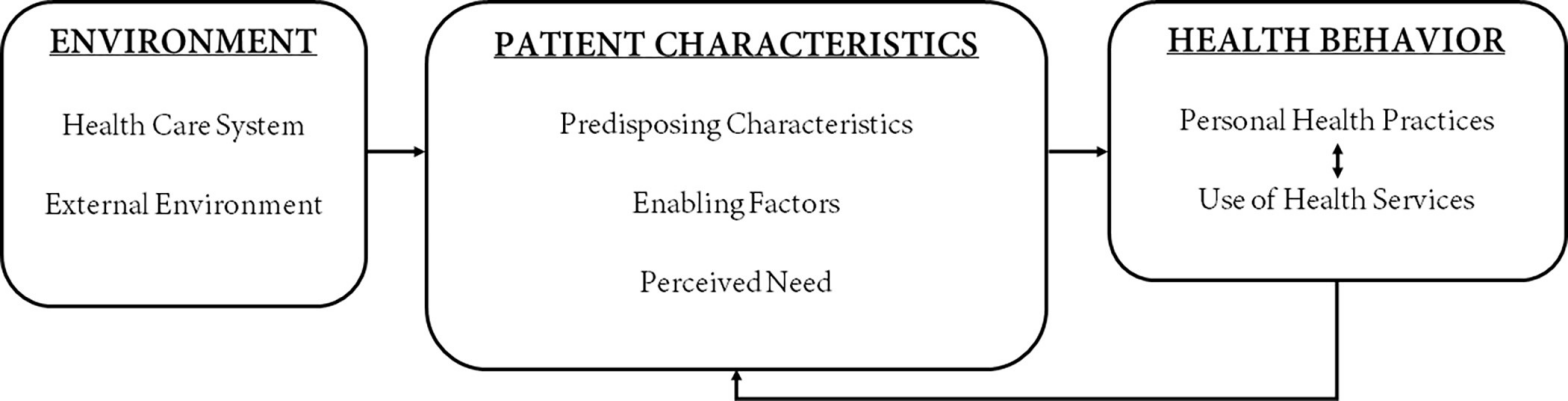
The Andersen’s behavioral model of health services use (adapted from Andersen) [15].

The ABMHSU provides a theoretical framework for understanding how patient and environmental factors impacts health behaviors and outcomes. These factors can be grouped into seven domains: patient characteristics (*predisposing characteristics*, *enabling factors*, perceived *need*), health care system environment (*system*, *clinic*, *provider*) and external environment (Fig 1). Multiple studies have used the ABMHSU to evaluate the use of health services among patients with chronic illness for purposes of identifying resolvable barriers to service utilizations in order to develop interventions to improve outcomes [16–18].

In this study, we define predisposing factors as those factors that influence decision-making about hypertension, including attitudes, knowledge, and perceived control. Enabling factors included having appropriate community and individual-level resources necessary for accessing care. The perceived needs domain was defined as how individuals view their own health and functional state, which can be influenced positively or negatively by their perceived severity of health. The health care environment entails primary elements of healthcare services that create the health care environment for the patient. Finally, the external environment includes physical, political, and economic factors unrelated to the health care environment.

### Study site and setting

This qualitative study was conducted as part of a larger study to develop an outpatient intervention to improve care among adults with hypertensive urgency. We conducted 38 qualitative interviews with three stakeholder groups relevant to the outpatient care for hypertensive urgency: 1) hypertensive urgency patients who have successfully attended the outpatient clinic; 2) hypertensive urgency patients who have not attended the clinic, and 3) healthcare workers. These interviews explored facilitators and barriers to outpatient clinic attendance and anti-hypertensive medication adherence. Data collection occurred between March and May 2020. The study received ethical approval from the Weill Cornell Medicine [Ref: 19–11021145] and the Tanzanian National Institute for Medical Research [Ref: NIMR/HQ/R.8a/Vol. IX/3349].

The study was conducted in the outpatient medical clinics of three hospital facilities in Mwanza City, Tanzania. The three study sites all together serve approximately 3000 adults living with hypertension annually. All clinics were following the national guidelines for treating hypertension [19]. Anti-hypertensive medications are widely available and inexpensive by high-income countries’ standards but can still be cost-prohibitive for most Tanzanians who live on <$100/month. Patients pay for services through a combination of insurance, government subsidy, and out-of-pocket contributions.

### Participants

Outpatients with hypertensive urgency were eligible to participate in the study if they were aged 18 years and above, fluent in Swahili, and able to provide informed consent. Patients with hypertension were categorized as not attending the outpatient clinic if they missed clinic appointments for >3 months. Healthcare workers were invited to participate in the study if they worked in the outpatient hypertension clinic at one of the three hospitals where participants were recruited.

### Procedures

Hypertensive urgency patients and healthcare workers were recruited at the clinic by the study team. Hypertensive urgency patients were selected focusing on their experience with hypertension urgency, either being a frequent attainder or non-attainders of the clinic. Non-attainders were identified from the previous cohort study of hypertensive patients conducted by RNP in the same hospital facilities of the current study. To establish non-attendance for >3 months, we assessed data on self-reported medication adherence and reviewed the medical record of clinic attendance. All participants in the previous cohort had contact information and provided their preferences on how they could be reached for future follow-up. Using the contact information extracted from patient files, the health worker contacted potential participants to provide brief information about the study.

All potential participants interested in joining the study were referred to the study team to receive additional information, confirm eligibility, and complete written informed consent procedures. Upon enrollment, participants were invited to schedule a time and choose their preferred location for the interview. Face-to-face interviews were conducted in Swahili, by trained Tanzanian researchers who had prior experience with qualitative research and had no affiliation with the clinic providing the hypertension care. Interviews took approximately one hour and were audio-recorded with the participant’s consent. Participants were given an incentive of 5000 Tanzanian Shillings (approximately $2.50) per interview. Following the completion of the interview, the audio files were transcribed and translated verbatim into English by research assistants fluent in both English and Swahili.

### Data collection instrument

The development of the interview guide was informed by the ABMHSU (see Fig 1) described above. The guide included open-ended questions and probes related to facilitators and barriers to hypertension care. Each section of the guide began with an opening question, followed by potential probes to be used to explore the topic in greater depth. Examples of questions include *“What are the things that have enabled you to come to your hypertension clinic visits and take your hypertensive medications?”*, *“What are the barriers you have faced attending your clinic appointments?”* and *“How do you think patients feel about services provided at the outpatient hypertension clinic?”* The guide was contextualized according to the patient’s status (i.e., attending or not attending clinic) and healthcare worker occupation (e.g., doctor, nurse). The semi-structured guides were piloted among three hypertensive patients and two healthcare workers prior to the commencement of data collection; data from these pilot interviews were not included in the analysis.

### Analysis

Data were analyzed using principles of applied thematic analysis [20] and consensual qualitative research [21]. Two team members [GAK, EO] independently reviewed transcripts to generate preliminary codes, which informed the development of a structured codebook focused on identifying the facilitators and barriers to clinic attendance and medication adherence. The 38 transcripts were uploaded to NVivo version 12 [22] and coded independently by two team members [OM, GAK] using the final codebook. After coding, code-level queries were run, analytic memos were written to synthesize the content and make comparisons across participant groups. These memos followed an established template of ABMHSU domains to extract and synthesize the core meaning from text related to each theme and to identify representative quotes. In writing memos, barriers and facilitators were analyzed as “barriers/facilitators” because participants’ discussions about clinic attendance and medication adherence behaviors often included their challenges and successes. At each step, coding team members [OM, GAK, and EO] returned to the original data to ensure that participants’ narratives and perspectives were retained. Disagreements were discussed until consensus was reached.

## Results

### Characteristic of study participants

A total of 38 participants participated in in-depth interviews; their ages ranged from 28 years to 77 years. An overview of the demographic information of the study participants can be found in Table 1.

**Table 1:** Participant’s socio-demographic characteristics.

Participants’ characteristics	Patient with Hypertension attending clinic (n = 13)	Patient with Hypertension not attending clinic (n = 11)	Healthcare workers (n = 14)
**Age,** *mean (range)*	56 (43–67)	54 (45–77)	42 (28–58)
**Gender**			
Female n (%)	8 (61%)	3 (27%)	11 (78%)
**Level of Education**			
Primary School n (%)	7 (54%)	7 (64%)	
Secondary school and above n (%)	6 (46%)	4 (36%)	14 (100%)
**Occupation** n (%)			
Employed by others	3 (23%)	3 (27%)	14 (100%)
Self Employed	10 (77%)	5 (45%)	-
Retired	-	1 (9%)	-
Home maker	-	2 (18%)	-
**Health Insurance cover n (%)**			
Insured	4 (31%)	1 (9%)	
Not insured	9 (69%)	10 (91%)	
**Cadre** n (%)			
Clinical assistants	N/A	N/A	2 (14%)
Nurses	N/A	N/A	8 (57%)
Doctors	N/A	N/A	4 (29%)

N/A: Not applicable

In their narrative, patients and healthcare workers discussed 21 types of barriers/facilitators to clinic attendance and medication adherence: 12 common to both behaviours, 6 distinct to clinic attendance, and 3 distinct to medication adherence (Table 2).

**Table 2:** Barriers/facilitators affecting clinic attendance and medication adherence.

Attendance alone	Attendance and adherence	Adherence alone
Transport to clinic	Traditional medicine	Drug stock
Clinic location	Knowledge and awareness of HTN	Medication side effects
Clinic appointment	Stigma	Medicine beliefs
Patient-provider interaction	Social support	
Service fragmentation	Health insurance	
Quality of care	Reminder cues	
	HTN symptoms	
	Self-efficacy	
	Peer support	
	Specialized care	
	Social services	
	Religious beliefs	

**Fig 2:**
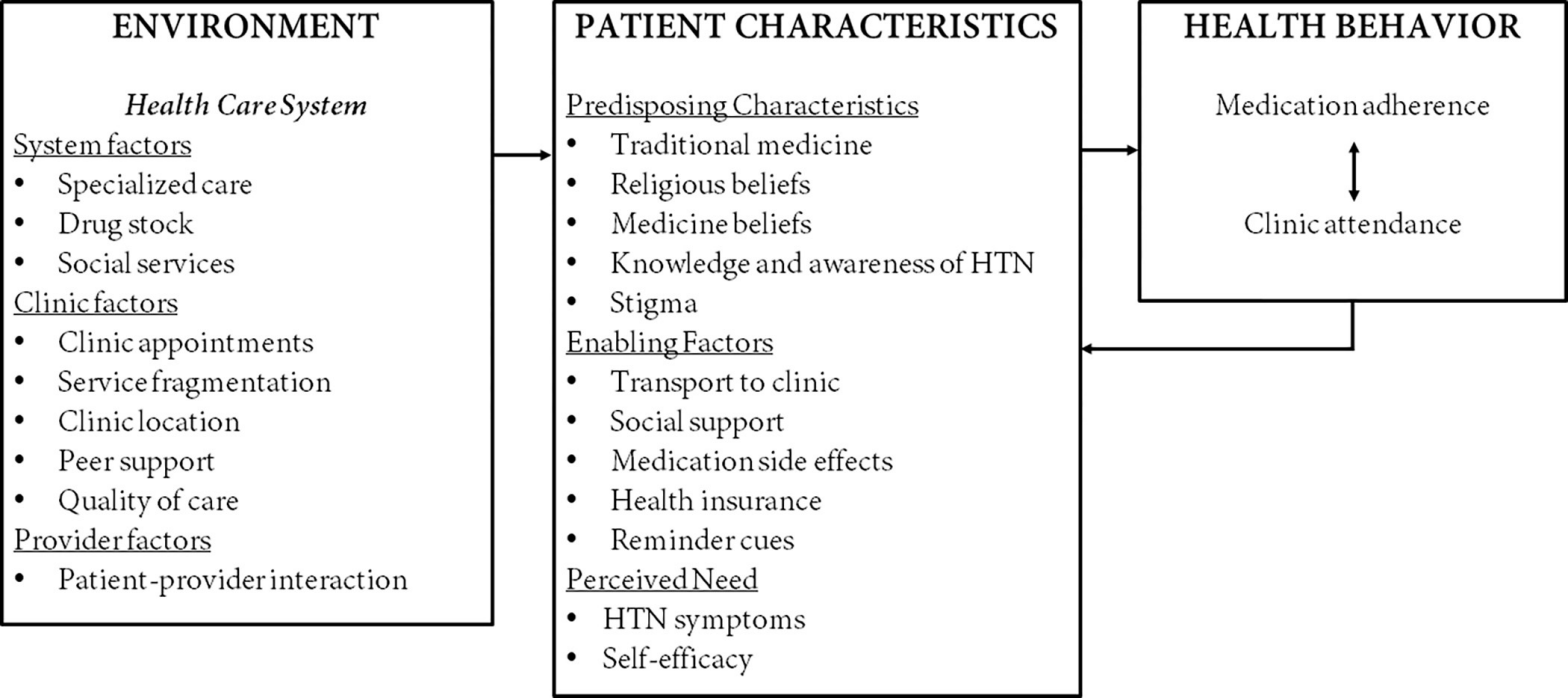
Barriers and facilitators for clinic attendance and medication adherence among hypertensive urgency patients in Tanzania.

Barriers/facilitators are presented based on the ABMHSU patient (predisposing characteristics, enabling factors, and perceived need) and environment (health system, clinic, and provider) domains (Fig 2). Tables 3 and 4 report barriers/facilitators with illustrative core ideas. S1 Table highlights detailed description of barriers and facilitators as identified by the study participants.

**Table 3:** Patient-level barriers/facilitators to clinic attendance and medication adherence organized according to the domains of the Andersen’s behavioral model of health services use.

ABMHSU domain	Barrier/facilitator	Selected patient quotes
Predisposing Characteristics	Traditional medicine	“Some people believe that a person can be bewitched to be hypertensive. Therefore, people who think like that go to the traditional healers to find medicine.”–M, A, C2
		“. . . they (patients) are trying to find cheaper treatments. To the traditional healer, patients pay once and get traditional medicine without any extra cost of clinic visits. So, to a patient, this is a relief.”–H, C2
	Religious beliefs	“A patient will tell you, "I won’t take medication. I will go to Pastor [name blinded]; he will pray for me."”–H, C1
		"In those churches, other people testify that ‘I was diagnosed with hypertension, but now I do not have hypertension because of prayers am cured,’ but this is not true."–H, C1
	Medicine beliefs	“I have observed that the traditional treatment is more effective than using drugs from the hospital because it manages blood pressure for an extended time.”–F, A, C1
		“. . . people are saying that if you take too much hospital medicine, it can poison the body. That is why people prefer using traditional medicine.–F, NA, C2
	Knowledge and awareness of HTN	“I think the community is not aware of the cause of hypertension.”–M, A, C2
		“We need to educate our patients. They need to have proper knowledge of severe hypertension, the signs and symptoms, and the treatments.”–H, C1
	Stigma	“. . . many are afraid to take hospital medications because they will be told they have HIV.”–F, A, C1
Enabling Factors	Social support	“My wife always encourages me to attend the clinic every month and take medications as prescribed.”–M, A, C2
		"I am grateful for them [children].. . . They remind me to take medicine if I do not remember."–M, A, C2
	Transport to clinic	“I need money for transport.. . . there are times I don’t attend the clinic because I don’t have money.”–F, A, C1
		“You may be willing to go to the clinic, but you have no money to cover transport.”–M, NA, C1
	Medication side effects	“I experience some problems when I take antihypertensive medications. I get headaches and feel dizzy.”–M, A, C2
		“There was a time when I took certain antihypertensive medicine, and I did not feel well; it made me sleep for a long time. I felt so weak.”–F, A, C2
	Health insurance	“Those in the health insurance scheme adhere to medications and have good clinic attendance compared to those not in the health insurance scheme.”–H, C2
		“Most patients not in the health insurance scheme cannot afford to pay for their medications, especially those with low income.”–H, C2
	Reminder cues	". . . around noon, we will call our patients to remind them that tomorrow is their clinic."–H, C1
Perceived Need	HTN symptoms	“Most of the patients will start medication after being hospitalized following hypertension complications …”–H, C1
		I am hypertensive, and I know that if I do not take medicine, it can reach a time I can fall down (have a stroke).–M, A, C2
	Self-efficacy	“I have my ability, and I have complete self-control in taking medications.”–F, A, C3
		"There is nothing beyond my control.. . . at first, I had much trouble taking the medications, but now I am used."–F, A, C1

M–male patient, F–female patient, A–Attending clinic, NA–Not attending clinic, C–outpatient clinic at (1: consultant hospital, 2: regional hospital, 3: district hospital), H–healthcare worker.

**Table 4:** Environmental barriers/facilitators to clinic attendance and medication adherence organized according to the domains of the Andersen’s behavioral model of health services use.

ABMHSU domain	Barrier/facilitator	Selected patient quotes
System factors	Specialized care	“… specialists should be increased because they better attend to patients when they are many.”–M, A, C2
	Drug stock	“… sometimes, they (patients) cannot access medications, or they can only get one drug at the hospital pharmacy.”–H, C2
		“Medications are often not available [at the hospital pharmacy], so they have to buy these medications at other pharmacies.”–H, C3
	Social services	"Social welfare officers evaluate the patient’s economic condition and provide an exemption certificate to help patients who cannot afford to pay for their treatment."–H, C2
		". . . they have exempt payment for all chronic diseases, so we do not pay for our treatments, including medications."–M, A, C3
Clinic factors	Clinic appointments	"The services from the clinic are time-consuming, and I still have other responsibilities to do."–M, NA, C2
	Service fragmentation	"At the clinic, there are many procedures that a patient has to go through before getting treatment."–M, NA, C2
	Clinic location	“I was referred to this health facility. . . It is very far [approximately 150 kilometers from the participant’s home].”–F, A, C1
	Peer support	"Patients who attend the clinic and who adhere to their medications can be used to educate other patients."–H, C1
	Quality of care	"We do receive good services, and doctors are available. If you come here, you receive good treatment on time."–F, A, C1
Provider factors	Patient-provider interaction	“. . . sometimes healthcare workers use harsh language/or are rude, so to avoid quarreling with them, I quit attending the clinic. . .”–M, NA, C2

M–male patient, F–female patient, A–Attending clinic, NA–Not attending clinic, C–outpatient clinic at (1: consultant hospital, 2: regional hospital, 3: district hospital), H–healthcare worker.

### Patient characteristics

#### Predisposing characteristics

Patients and healthcare workers reported five barriers/facilitators under the predisposing characteristics domain. These barriers/facilitators were traditional medicine, religious beliefs, medicine beliefs, knowledge and awareness of hypertension, and stigma. Among these, traditional medicines were frequently reported as an alternative to allopathic medicine by patients, and they were identified as a critical barrier to clinic attendance and medication adherence by healthcare workers. In this context, we refer to traditional medicine as remedies made from plants prescribed by traditional healers. The preferential use of traditional medicine was primarily driven by the belief that hypertension is caused by witchcraft. Also, traditional medicines are low-priced and could be used for a short period compared to expensive, lifelong allopathic medicine. In this manner, traditional medicines were perceived as curative for hypertension instead of allopathic medicines that are lifelong. Furthermore, some patients reported that traditional medicines were more accessible and perceived to be more efficacious than allopathic medicine.

Most healthcare providers pointed out that solid religious belief in healing hypertension by prayer was a barrier to clinic attendance and medication adherence. Because of this belief, patients with hypertension were likely to stop attending clinics and quit using medicines prescribed by their healthcare providers.

Some patients described medicine beliefs as a barrier to medication adherence. It was believed that long-term use of hospital medicine is harmful and could result in adverse health outcomes. For example, it was generally believed that diabetes in people with hypertension was directly linked to the longstanding use of antihypertensive medicine. This belief was linked to some patients’ decisions to use traditional medicine.

Healthcare workers discussed proper knowledge and hypertension awareness as important barriers/facilitators to clinic attendance and medication adherence. Patients with poor knowledge of the cause, symptoms, and treatment of hypertension were observed to have poor clinic attendance and medication adherence. Nevertheless, healthcare workers noted that patients who are aware of their hypertension status have excellent records on clinic attendance and report good adherence to anti-hypertensive medicine.

Although patients were aware of the importance of adhering to hypertensive medication, they pointed out the stigma attached to taking lifelong medicine. According to patients, the public perception of taking medication daily was associated with the treatment of HIV, making patients reluctant to attend a monthly clinic and adhere to the medication regimen.

#### Enabling factors

According to interviewed patients and healthcare workers, five facilitators/barriers were designated to the enabling factors domain. These barriers/facilitators were social support, transport to clinic, health insurance, medication side effects, and reminder cues. The paragraphs below provide a brief description of each barrier/facilitator.

Most patients reported that social support facilitated clinic attendance and medication adherence. This support was mainly from their close family members such as a spouse, children, and other immediate relatives. The assistance from these family members was likely to be available when a patient can disclose their hypertension status to the family. Social supporters reminded the patient when to attend the clinic and take medication, escorted the patient to the clinic whenever needed, and provided financial and moral support.

Some patients, especially those living far from the outpatient clinic, reported the cost of traveling to and from the clinic as a significant barrier to clinic attendance. Patients without financial support could not navigate this challenge.

Both patients and healthcare workers noted that health insurance subscription was a critical facilitator to clinic attendance and medication adherence. The cost of treatment (i.e., consultation and investigation fees, medicine prices) was entirely covered by the health insurance. Thus, patients with insurance cover do not bear the burden of treatment costs, unlike patients without insurance subscriptions, whom most could not afford.

While hypertension can be safely treated in most individuals, side effects of drugs are relatively frequent and could have notable effects on quality of life. A few patients shared their experiences regarding the side effects of antihypertensive medicines that led to poor medication adherence.

Reminder cues were reported by patients and healthcare workers as a facilitator to clinic attendance and medication adherence. Patients used several methods to remember taking antihypertensive medicines, including setting alarms on mobile phones and putting pillboxes at the bedside. Similarly, healthcare workers called patients a day before to remind them about the upcoming clinic appointment.

#### Perceived need

The analysis of the perceived need domain revealed two barriers/facilitators, including hypertension symptoms and perceived self-efficacy (i.e., belief about one’s ability to handle hypertension). According to healthcare providers, most patients diagnosed with hypertension deny the diagnosis mainly due to a lack of symptoms. As a result, most patients will not attend the clinic or take antihypertensive medicine because they believe they are not sick. On the contrary, patients who accepted the diagnosis and believed that they could handle hypertension reported attending the clinic regularly and had good adherence.

### Health care environment

#### System factors

Patients in our study identified three services that impacted their health care environment. These services were specialized care, drug stock, and social welfare services. The majority of the patients emphasized that services provided by specialized doctors enhanced clinic attendance and medication adherence. However, not all clinics had enough specialized doctors to attend to overflowing patients per clinic. Furthermore, patients observed that antihypertensive drugs were often out of stock at public clinics, where they are sold at a subsidized price. Therefore, the drug stock affected medication adherence, especially for those who could not afford to purchase drugs at private pharmacies. Lastly, the availability of social welfare services at the clinic facilitated clinic attendance and medication adherence. Among the services provided under the social welfare services were cost exemption for those who could afford treatment costs and a pre-identified group of patients or diseases.

#### Clinic factors

Patients’ and healthcare workers’ narratives highlighted five clinic barriers/facilitators; clinic appointment, service fragmentation, clinic location, quality of care, and peer support. All of these barriers/facilitators, except peer support, were linked to clinic attendance alone. Most patients were given monthly clinic appointments for clinical evaluation and drug refills. Due to a shortage of healthcare workers, patients reported spending a significant time in the clinic, which affected their routine. Furthermore, service fragmentation at the clinic made it hard for patients to complete clinic appointments in time, especially unaccompanied elderly patients. Patients felt that organizing all services at one point would expedite service provision. A few patients reported that quality of care enhanced their clinic attendance. This observation was evidently among patients with health insurance at some clinics where patients with insurance were given differentiated care. Healthcare providers felt that incorporating peer support programs at the standard of care would increase clinic attendance and medication adherence. Thus, patients who had hypertensive urgency and managed to get blood pressure under control would be used to provide education to their peers.

#### Provider factors

Some patients encountered incompetent health care providers who were incapable of meeting their needs during the monthly clinic visit. This interaction with incompetent providers resulted in the negative experience of using health care services available in the clinic. For example, patients mentioned that some health care providers are using offensive language and ignore their questions about hypertension. For some patients, this experience discouraged them from continuing to attend clinic appointments.

## Discussion

Hypertensive urgency is common in clinics in Africa, and over 1/3 of adults with hypertensive urgency will either be hospitalized or die within one year after presenting with hypertensive urgency. Hospitalization and death in patients with hypertensive urgency are strongly linked with poor clinic attendance and poor medication adherence. This study used qualitative methods guided by ABMHSU to explore barriers/ facilitators to clinic attendance and medication adherence among adults with hypertension urgency. This study represents a necessary step in contextualizing barriers/facilitators to clinic attendance and medication adherence among adults with hypertension urgency in Mwanza to develop a context-appropriate intervention for this group.

Our data reflects 21 barriers/facilitators to clinic attendance and medication adherence among patients with hypertensive urgency. The majority (12 out of 21) of the barriers/facilitators affected both behaviors. This overlap of barriers/facilitators across behaviors in the care continuum has been observed in several studies that assessed barriers and facilitators to chronic disease care engagement [16, 17]. However, it is worth noting that the magnitude of the effect of the barrier/facilitator often differs between behaviors. For example, traditional medicine hampered medication adherence more than clinic attendance in this study. This finding may be useful in the design of intervention so that targeting a set of facilitators/barriers might improve clinic attendance and medication adherence. Nevertheless, there should be a recognition that some facilitators/barriers are related to only one behavior, such as medication side effects that affect adherence alone.

Findings from the current study show that beliefs about traditional medicines can act as barriers to clinic attendance and medication adherence. In this study, participants’ beliefs about traditional medicine’s availability, accessibility, and efficacy influenced help-seeking behavior for hypertension. Similar findings have been reported by previous studies of patients with hypertension in Tanzania, Columbia, South Africa and Nepal [23–26]. Such finding shows the importance of asking about the history of the use of alternative, traditional, or supplemental therapy from patients during the clinical encounter as a missed opportunity to address not only the underlying beliefs in the efficacy of traditional medicine but also to further counsel the patients about the nature and course of HTN.

Fear about the effect of poisoning from long-term use of hospital medicine for hypertension was reported to affect medication adherence. A significant correlation between beliefs about medication was found among hypertensive patients in Ghana and Nigeria [27], with patients who were worried about the adverse effects of antihypertensive drugs less likely to be adherent to their medications. Qualitative studies in Pakistan [28], Malaysia [29], and Nigeria [30] found that lack of faith in hospital medicine and perceived side effects hindered adherence to antihypertensive medication. Improving patients’ knowledge and attitudes to hospital medications may empower patients to be more concerned about their health and hence more involved in their treatment.

The health providers and patients in this study reported the ability to afford HTN treatment as a significant barrier to medication adherence. For patients living below the poverty line, the cost of purchasing antihypertensive poses a substantial burden to themselves and their dependents. According to our study participants’ narratives, this burden triggers stress and affects their general wellbeing. Consultation fee and treatment cost exemption, having health insurance were identified as important facilitators. These findings are consistent with other studies [26, 30, 31]. Participants in our study also pointed out the distance to a health facility as an important barrier to clinic attendance and adherence to antihypertensive medication. Longer distance to health facility contributed to non-adherence of hypertensive patients in Ethiopia [32].

In our study, participants identified lack of symptoms as one of the reasons people with hypertensive urgency may not attend the clinic or take hypertensive medications. Studies in Nigeria, India and Congo have shown that the absence of symptoms significantly contributed to poor compliance to hypertensive therapy [33–36].

Our data shows that lack of both emotional and material support was a major barrier to clinic attendance and medication adherence among people with hypertension urgency. Other studies have reported that an absence of family support has a strong negative effect on adherence among hypertensive patients in Ethiopia [37] and Nigeria [38]. Likewise, studies in Congo reported that patients who received support from family members, and particularly reminders about taking their medications, were more likely to be adherent to their antihypertensive medications [35, 39].

Health systems factors, including lack of specialists, cost of medication, length of the clinic visit, and the fragmented nature of services offered, were among the critical barriers to clinic attendance. Weak health systems have been identified as a significant obstacle in effectively responding to the rising burden of chronic conditions such as hypertension in developing countries [40]. Calls have been made to recognize and analyze the complex interactions between health systems and their effects on hypertension management in developing countries [41]. Factors such as confidence in the physician’s knowledge or ability have been found to be significantly related to medication adherence in developed countries. Studies have found dissatisfaction with the health services and treatment providers influenced adherence significantly among patients with hypertension [42, 43]. Hypertensive individuals who were satisfied with the care received are more likely to adhere to the proposed medication treatment. Inconvenient clinic operating hours, long waiting times have also been found to be inhibitors of adherence [44, 45].

In our study, traditional medicine was among the salient barriers to clinic attendance and medication adherence. Prospective intervention designs could consider engaging with traditional healers or educating traditional healers to improve adherence for people with hypertension in communities where concurrent traditional medicine use is common. Previous studies [46–50] demonstrate that partnerships with traditional healers can improve health education for chronic diseases.

### Strengths and limitations

This was a qualitative study aimed at examining context-specific barriers and facilitators to hypertension clinic attendance and adherence to antihypertension. The use of open-ended questions helped to gather rich information on participants’ views and experiences. Our participants included both patients attending and those that had defaulted on regular clinic attendance and medication as well as health service providers. Such triangulation of data sources helped provide an in-depth understanding of context-specific barriers and facilitators from different perspectives. However, some study limitations should be considered, including the exploratory nature of this qualitative study, and confinement to a small sample of patients and health workers in three health facilities limit the generalizability of our findings to other population.

## Conclusion

The current study identified 21 barriers/facilitators to clinic attendance and medication adherence among patients with hypertensive urgency. The barriers to clinic attendance and medication adherence may be surmountable using tailored supportive intervention approaches, such as peer counsellors, to help patients overcome social challenges of clinic attendance and medication adherence.

## Supporting information

S1 Table Barriers/facilitators to clinic attendance and medication adherence, categorized by Andersen’s behavioral model of health services use domains. (DOCX)
