# Supplementary material for: Understanding barriers and facilitators to clinic attendance and medication adherence among adults with hypertensive urgency in Tanzania
Source: PLOS Glob Public Health. 2022 Aug 23;2(8):e0000919. doi: 10.1371/journal.pgph.0000919 (PMC10021323; doi:10.1371/journal.pgph.0000919)
Supplement: S1 Table — (DOCX) [file pgph.0000919.s001.docx]

**S1 Table**. Barriers/facilitators to clinic attendance and medication adherence, categorized by Andersen’s behavioral model of health services use domains

|  | **Barriers** | **Facilitators** |
| --- | --- | --- |
| *Patient characteristics* | | |
| Predisposing Characteristics | Traditional medicines perceived as efficacious | Having knowledge of hypertension |
|  | Hospital medicine perceived as poisonous |  |
|  | Belief in healing by prayer |  |
|  | Low knowledge and awareness of hypertension |  |
|  | Stigma of long-term allopathic medication use |  |
| Enabling Factors | Lack of social support | Having health insurance |
|  | Lack of transport fare to clinic | Reminder cues |
|  | Medication side effects |  |
|  | inability to pay for hypertension care |  |
| Perceived Need | Having no disruptive hypertension symptoms | One’s ability to handle hypertension |
| *Health care environment* | | |
| System factors | Lack of specialized care | Provision of social services at clinic |
|  | Stock out medicines |  |
| Clinic factors | Long waiting time at the clinic | Availability of peer support |
|  | Distant clinic facility | Quality care |
|  | Fragmented services at the clinic |  |
| Provider factors | Negative attitudes among healthcare workers | Affirming interaction |
